# Racial and Ethnic Disparities in Rates of COVID-19–Associated Hospitalization, Intensive Care Unit Admission, and In-Hospital Death in the United States From March 2020 to February 2021

**DOI:** 10.1001/jamanetworkopen.2021.30479

**Published:** 2021-10-21

**Authors:** Anna M. Acosta, Shikha Garg, Huong Pham, Michael Whitaker, Onika Anglin, Alissa O’Halloran, Jennifer Milucky, Kadam Patel, Christopher Taylor, Jonathan Wortham, Shua J. Chai, Pam Daily Kirley, Nisha B. Alden, Breanna Kawasaki, James Meek, Kimberly Yousey-Hindes, Evan J. Anderson, Kyle P. Openo, Andrew Weigel, Maya L. Monroe, Patricia Ryan, Libby Reeg, Alexander Kohrman, Ruth Lynfield, Erica Bye, Salina Torres, Yadira Salazar-Sanchez, Alison Muse, Grant Barney, Nancy M. Bennett, Sophrena Bushey, Laurie Billing, Eli Shiltz, Melissa Sutton, Nasreen Abdullah, H. Keipp Talbot, William Schaffner, Jake Ortega, Andrea Price, Alicia M. Fry, Aron Hall, Lindsay Kim, Fiona P. Havers

**Affiliations:** 1 COVID-19-Associated Hospitalization Surveillance Network, US Centers for Disease Control and Prevention, Atlanta, Georgia; 2 US Public Health Service, Rockville, Maryland; 3 General Dynamics Information Technology, Atlanta, Georgia; 4 California Emerging Infections Program, Oakland; 5 Colorado Department of Public Health and Environment, Denver; 6 Connecticut Emerging Infections Program, Yale School of Public Health, New Haven; 7 Department of Pediatrics, Emory University School of Medicine, Atlanta, Georgia; 8 Department of Medicine, Emory University School of Medicine, Atlanta, Georgia; 9 Georgia Emerging Infections Program, Georgia Department of Health, Atlanta; 10 Atlanta Veterans Affairs Medical Center, Atlanta, Georgia; 11 Iowa Department of Public Health, Des Moines; 12 Maryland Department of Health, Baltimore; 13 Michigan Department of Health and Human Services, Lansing; 14 Minnesota Department of Health, St Paul; 15 New Mexico Department of Health, Santa Fe; 16 New York State Department of Health, Albany; 17 University of Rochester School of Medicine and Dentistry, Rochester, New York; 18 Ohio Department of Health, Columbus; 19 Oregon Health Authority, Portland; 20 Vanderbilt University School of Medicine, Nashville, Tennessee; 21 Salt Lake County Health Department, Salt Lake City, Utah; 22 Career Epidemiology Field Officer, US Centers for Disease Control and Prevention, Atlanta, Georgia

## Abstract

**Importance:**

Racial and ethnic minority groups are disproportionately affected by COVID-19.

**Objectives:**

To evaluate whether rates of severe COVID-19, defined as hospitalization, intensive care unit (ICU) admission, or in-hospital death, are higher among racial and ethnic minority groups compared with non-Hispanic White persons.

**Design, Setting, and Participants:**

This cross-sectional study included 99 counties within 14 US states participating in the COVID-19–Associated Hospitalization Surveillance Network. Participants were persons of all ages hospitalized with COVID-19 from March 1, 2020, to February 28, 2021.

**Exposures:**

Laboratory-confirmed COVID-19–associated hospitalization, defined as a positive SARS-CoV-2 test within 14 days prior to or during hospitalization.

**Main Outcomes and Measures:**

Cumulative age-adjusted rates (per 100 000 population) of hospitalization, ICU admission, and death by race and ethnicity. Rate ratios (RR) were calculated for each racial and ethnic group compared with White persons.

**Results:**

Among 153 692 patients with COVID-19–associated hospitalizations, 143 342 (93.3%) with information on race and ethnicity were included in the analysis. Of these, 105 421 (73.5%) were 50 years or older, 72 159 (50.3%) were male, 28 762 (20.1%) were Hispanic or Latino, 2056 (1.4%) were non-Hispanic American Indian or Alaska Native, 7737 (5.4%) were non-Hispanic Asian or Pacific Islander, 40 806 (28.5%) were non-Hispanic Black, and 63 981 (44.6%) were White. Compared with White persons, American Indian or Alaska Native, Latino, Black, and Asian or Pacific Islander persons were more likely to have higher cumulative age-adjusted rates of hospitalization, ICU admission, and death as follows: American Indian or Alaska Native (hospitalization: RR, 3.70; 95% CI, 3.54-3.87; ICU admission: RR, 6.49; 95% CI, 6.01-7.01; death: RR, 7.19; 95% CI, 6.47-7.99); Latino (hospitalization: RR, 3.06; 95% CI, 3.01-3.10; ICU admission: RR, 4.20; 95% CI, 4.08-4.33; death: RR, 3.85; 95% CI, 3.68-4.01); Black (hospitalization: RR, 2.85; 95% CI, 2.81-2.89; ICU admission: RR, 3.17; 95% CI, 3.09-3.26; death: RR, 2.58; 95% CI, 2.48-2.69); and Asian or Pacific Islander (hospitalization: RR, 1.03; 95% CI, 1.01-1.06; ICU admission: RR, 1.91; 95% CI, 1.83-1.98; death: RR, 1.64; 95% CI, 1.55-1.74).

**Conclusions and Relevance:**

In this cross-sectional analysis, American Indian or Alaska Native, Latino, Black, and Asian or Pacific Islander persons were more likely than White persons to have a COVID-19–associated hospitalization, ICU admission, or in-hospital death during the first year of the US COVID-19 pandemic. Equitable access to COVID-19 preventive measures, including vaccination, is needed to minimize the gap in racial and ethnic disparities of severe COVID-19.

## Key Points

**Question:**

Are rates of COVID-19–associated hospitalization, intensive care unit (ICU) admission, or in-hospital death higher among individuals who belong to racial and ethnic minority groups compared with those who identify as non-Hispanic White?

**Findings:**

In this cross-sectional study of 143 342 individuals hospitalized with COVID-19, non-Hispanic American Indian or Alaska Native, Hispanic or Latino, non-Hispanic Black, and non-Hispanic Asian or Pacific Islander persons were more likely to have a COVID-19-associated hospitalization, ICU admission, or in-hospital death compared with non-Hispanic White individuals during the first year of the pandemic.

**Meaning:**

In this study, US residents who belong to racial and ethnic minority groups experienced severe COVID-19–associated outcomes disproportionately; equitable access to preventive measures, such as COVID-19 vaccines, is needed for these populations.

## Teaser

This cross-sectional study evaluates whether rates of COVID-19–associated hospitalization, intensive care unit admission, or in-hospital death are higher among racial and ethnic minority groups compared with non-Hispanic White persons.

## Introduction

The coronavirus disease 2019 (COVID-19) pandemic has disproportionately affected racial and ethnic minority populations in the United States, who are at an increased risk of infection, hospitalization, and death.^1,2^ During the first 4 months of the pandemic, data from the US Centers for Disease Control and Prevention (CDC) COVID-19–Associated Hospitalization Surveillance Network (COVID-NET)^3,4,5^ and other studies demonstrated that non-Hispanic Black persons were disproportionately hospitalized with COVID-19 and that racial and ethnic minority populations, including non-Hispanic Black and Hispanic or Latino persons, had higher rates of hospitalization compared with non-Hispanic White persons.^6,7,8^ Nonetheless, data on severe COVID-19 illness among other racial and ethnic minority groups, especially non-Hispanic American Indian or Alaska Native and non-Hispanic Asian or Pacific Islander populations, and longitudinal data across all racial and ethnic groups are limited. Using data from COVID-NET, a large, geographically diverse surveillance network for COVID-19–associated hospitalizations, we describe rates of hospitalization, intensive care unit (ICU) admission, and in-hospital death by race and ethnicity during the first year of the pandemic.

## Methods

COVID-NET surveillance activities were reviewed by CDC and were conducted consistent with applicable federal law and CDC policy (eg, 45 CFR. part 46.102(l)(2), 21 CFR part 56; 42 USC. §241(d); 5 USC §552a; 44 USC §3501 et seq). Sites participating in COVID-NET obtained approval from their respective state and local institutional review boards, as applicable. The requirement for informed consent was waived per 45 CFR 46. This cross-sectional study is reported following the Strengthening the Reporting of Observation Studies in Epidemiology (STROBE) reporting guidelines.

### COVID-NET Surveillance

COVID-NET, which has been previously described, conducts population-based surveillance for laboratory-confirmed COVID-19–associated hospitalizations among persons of all ages in 99 counties in 14 states (California, Colorado, Connecticut, Georgia, Iowa, Maryland, Michigan, Minnesota, New Mexico, New York, Ohio, Oregon, Tennessee, and Utah) and represents approximately 10% of the US population.^3^ Hospitalized residents in the COVID-NET catchment area who have a positive SARS-CoV-2 molecular or rapid antigen detection test during hospitalization or within 14 days prior to hospital admission are included in surveillance.

Trained surveillance staff identify persons hospitalized with laboratory-confirmed COVID-19 using laboratory, hospital, and reportable condition databases. Using a standardized data collection form, staff abstract medical records on a sample of patients to obtain data on demographic and clinical characteristics, underlying medical conditions, and clinical interventions and outcomes, including ICU admission, invasive mechanical ventilation (IMV), vasopressors, kidney replacement therapy (KRT), median length of stay (LOS) in the hospital, and in-hospital death from all causes.

For this analysis, we categorized race and ethnicity according to the National Center for Health Statistics (NCHS) categories^9^ as follows: Hispanic or Latino (Latino), non-Hispanic American Indian or Alaska Native (American Indian or Alaska Native), non-Hispanic Asian or Pacific Islander (Asian or Pacific Islander), non-Hispanic Black (Black), and non-Hispanic White (White). People who identified as more than 1 race and ethnicity or unknown race and ethnicity are captured by surveillance but were not included due to small numbers. If race was unknown but ethnicity was Latino, the person was classified as Latino. If ethnicity was unknown, non-Latino ethnicity was assumed. Race and ethnicity data were obtained from multiple sources, including notifiable disease, laboratory, and hospital databases. In most cases, race and ethnicity are self-reported, but the source could not be confirmed in every case.

### COVID-NET Sampling and Weighting Methodology

A minimum data set (including age, sex, race and ethnicity, surveillance site, hospital admission date, and positive SARS-CoV-2 test result and date) is reported for all persons identified by COVID-NET to produce weekly hospitalization rates stratified by age and race and ethnicity.^10^ An age- and surveillance site–stratified random sampling scheme is used to collect detailed clinical data for a representative sample of hospitalized adult patients aged at least 18 years; children younger than 18 years are sampled at a rate of 100%. The age strata used for sampling among adults are as follows: 18 to 49 years, 50 to 64 years, and 65 years or older. The sample size is powered to achieve a relative standard error of less than 30% for point estimates with values equal to or greater than approximately 10%, resulting in a 16% sampling rate for adult patients during the analytic time period. Sample weights are calculated as the inverse probability of being selected within each COVID-NET site and age group. These weights are adjusted for nonresponse, raked to adjust the sampled population to the total population using published procedures,^11^ and trimmed to reduce variability.

### Estimation of Population-Based Rates of COVID-19–Associated Hospitalization, ICU Admission, and In-Hospital Death

Cumulative and monthly COVID-19–associated hospitalization rates per 100 000 population, stratified by race and ethnicity, were calculated using all hospitalized persons in COVID-NET with known race and ethnicity for the numerator and NCHS vintage 2019 bridged-race population estimates for the denominator.^12^ Cumulative rates of ICU admission and in-hospital death were similarly calculated; however, because ICU admission and in-hospital death status were only available for sampled hospitalized patients, weighted frequencies of ICU admission and in-hospital death among sampled patients were used as the numerator. Both crude and age-adjusted rates were estimated. Age-adjusted rates accounted for differences in age distributions within race and ethnicity strata in the COVID-NET catchment area using the following age strata for adjustments: 0 to 17 years, 18 to 49 years, 50 to 64 years, 65 to 74 years, 75 to 84 years, and 85 years and older. Hospitalization, ICU admission, and in-hospital death rate ratios (RRs) for each racial and ethnic group were calculated in comparison with White persons.

### Statistical Analysis

Data from all patients hospitalized with COVID-19 during March 1, 2020, to February 28, 2021, were used to describe the demographic characteristics (age, sex, race and ethnicity) of hospitalized patients and hospitalization rates by race and ethnicity. All other analyses were limited to sampled hospitalized patients for whom medical record abstractions were completed and a discharge disposition was known. The weighted distributions of clinical characteristics and outcomes among hospitalized patients were calculated by age group and race and ethnicity; weighted percentages and unweighted case counts are presented throughout.

Data for sampled persons were analyzed using SAS survey procedures to account for sampling weights. *P* values for cumulative and monthly hospitalization rates, ICU admission, and in-hospital death rates and RRs were calculated using a Z test for the equality of 2 proportions. We calculated 95% CIs around rates and RRs assuming a simple random sample design and a normal distribution using the SAS STDRATE procedure with direct standardization. Statistical significance was set at α = .05, and all tests were 2-tailed. All analyses were performed using SAS version 9.4 (SAS Institute).

## Results

### Characteristics of All Hospitalized Patients Identified Through COVID-NET

From March 1, 2020, through February 28, 2021, 153 692 COVID-19–associated hospitalizations were reported to COVID-NET. A total of 10 350 hospitalizations (6.7%) were excluded due to the following: unknown race and ethnicity (8085 [5.3%]), race and ethnicity yet to be collected (1750 [1.1%]), and people of more than 1 race (515 [0.3%]). A total of 143 342 patients (93.2%) had race or ethnicity data and were included in analysis of hospitalization rates. Of the 143 342 hospitalizations, race was available but ethnicity was missing for 1230 (0.9%) or unknown for 7257 (5.1%); these were categorized as non-Latino. Of the 143 342 hospitalizations, ethnicity was available but race was missing for 61 (0.04%) or unknown for 16 913 (11.8%); all were of Latino ethnicity and were categorized as Latino.

Of all included hospitalizations, 105 421 (73.5%) were among patients aged 50 years or older, and 72 159 (50.3%) were male. The racial and ethnic distribution included 2056 (1.4%) American Indian or Alaska Native patients, 7737 (5.4%) Asian or Pacific Islander patients, 40 806 (28.5%) Black patients, 28 762 (20.1%) Latino patients, and 63 981 (44.6%) White patients (eTable 1 in the Supplement). Median (IQR) age by race and ethnicity was 55.0 (40.9-67.1) years for American Indian or Alaska Native patients, 61.6 (47.1-73.7) years for Asian or Pacific Islander patients, 59.2 (46.0-70.6) years for Black patients, 50.5 (36.9-63.9) years for Latino patients, and 69.5 (57.1-79.9) years for White patients.

### Demographic and Clinical Characteristics of a Weighted Sample of Hospitalized Patients

A total of 25 414 hospitalized patients were sampled for detailed medical record abstraction; of these patients, 25 281 (99.5%) with completed medical record review and a discharge disposition were included in analyses of clinical data. Demographic characteristics of sampled patients were like that of all hospitalized patients in COVID-NET (eTable 1 in the Supplement). Most sampled hospitalized patients had at least 1 underlying medical condition: American Indian or Alaska Native (90.0%; 95% CI, 84.3%-95.6%), Asian or Pacific Islander (88.9%; 95% CI, 86.4%-91.5%), Black (94.4%; 95% CI, 93.5%-95.4%), Latino (82.1%; 95% CI, 80.3%-83.9%), and White (94.4%; 95% CI, 93.6%-95.2%). The 3 conditions with highest prevalence were hypertension, obesity, and diabetes, with variations by race and ethnicity as well as age group (eTable 2 in the Supplement).

### Population-Based Rates of Hospitalization, ICU Admission and In-Hospital Mortality

**Figure 1.**
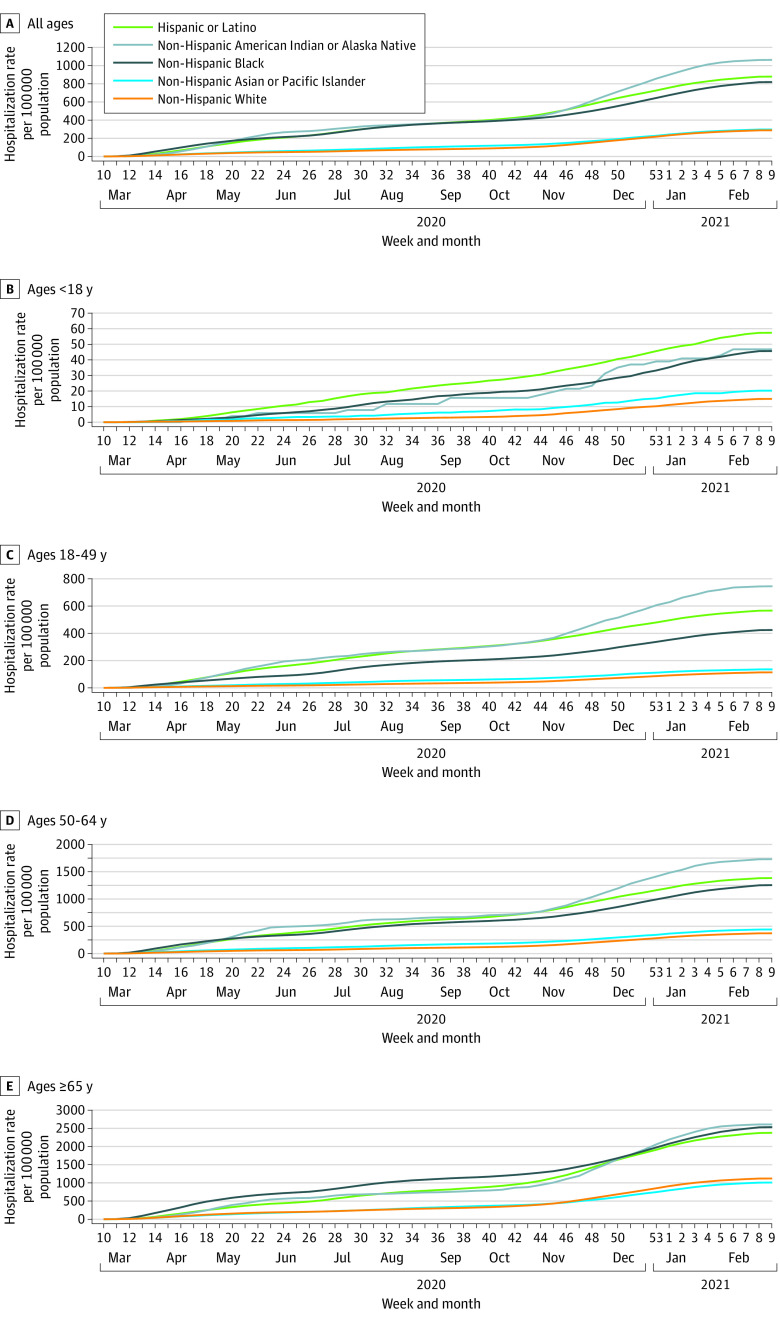
Cumulative COVID-19–Associated Hospitalization Rates, by Age Group and Race and Ethnicity, United States, March 1, 2020, to February 28, 2021 A, Cumulative hospitalization rates were age adjusted. Data are from the COVID-19-Associated Hospitalization Surveillance Network.

Cumulative age-adjusted hospitalization rates were highest among American Indian or Alaska Native, Latino, and Black persons (Figure 1A). Compared with White persons, cumulative age-adjusted hospitalization RRs were 3.70 (95% CI, 3.54-3.87) for American Indian or Alaska Native persons, 3.06 (95% CI, 3.01-3.10) for Latino persons, and 2.85 (95% CI, 2.81-2.89) for Black persons (Figure 1A and Table 1). Although hospitalization rates increased with age across all racial and ethnic groups, the highest hospitalization rates for each age group varied by race and ethnicity (Figure 1B-E). Latino persons had the highest cumulative hospitalization rates among children younger than 18 years (57.4 per 100 000 population vs 14.9 per 100 000 population for White persons). American Indian or Alaska Native persons had the highest rates among adults aged 18 to 49 years (744.3 per 100 000 population vs 113.5 per 100 000 population for White persons) and 50 to 64 years (1725.4 per 100 000 population vs. 370.4 per 100 000 population for White persons). American Indian or Alaska Native (2607.6 per 100 000 population), Black (2533.8 per 100 000 population), and Latino (2376.9 per 100 000 population) persons had the high rates among adults aged 65 years and older.

**Table 1.** Cumulative Rates and RRs of COVID-19–Associated Hospitalization, ICU Admission, and In-Hospital Death by Age Group and Race and Ethnicity, United States, March 1, 2020, to February 28, 2021

Outcome	Hispanic or Latino		Non-Hispanic							
	Rate (95% CI) per 100 000 population^a^	RR (95% CI)	American Indian or Alaska Native		Black		Asian or Pacific Islander		White	
			Rate (95% CI) per 100 000 population^a^	RR (95% CI)	Rate (95% CI) per 100 000 population^a^	RR (95% CI)	Rate (95% CI) per 100 000 population^a^	RR (95% CI)	Rate (95% CI) per 100 000 population^a^	RR (95% CI)
**All ages^b^**										
Hospitalization rate	879.2 (868.2-890.2)	3.06 (3.01-3.10)	1063.0 (1016.2-1109.9)	3.70 (3.54-3.87)	819.2 (811.1-827.3)	2.85 (2.81-2.89)	296.9 (290.2-303.6)	1.03 (1.01-1.06)	287.5 (285.3-289.7)	1 [Reference]
ICU admission rate	252.9 (246.9-258.9)	4.20 (4.08-4.33)	390.4 (361.4-419.5)	6.49 (6.01-7.01)	190.7 (186.7-194.6)	3.17 (3.09-3.26)	114.7 (110.5-118.9)	1.91 (1.83-1.98)	60.2 (59.1-61.2)	1 [Reference]
In-hospital death rate	124.5 (120.0-129.1)	3.85 (3.68-4.01)	232.8 (208.8-256.7)	7.19 (6.47-7.99)	83.6 (80.9-86.3)	2.58 (2.48-2.69)	53.2 (50.3-56.1)	1.64 (1.55-1.74)	32.4 (31.7-33.1)	1 [Reference]
**<18 y**										
Hospitalization rate	57.4 (53.5-61.2)	3.85 (3.45-4.29)	46.7 (28.0-65.4)	3.13 (2.08-4.72)	45.6 (42.2-49.1)	3.06 (2.73-3.43)	20.2 (16.6-23.8)	1.35 (1.11-1.65)	14.9 (13.6-16.2)	1 [Reference]
ICU admission rate	13.0 (11.1-14.8)	3.52 (2.82-4.40)	15.6 (4.8-26.4)	4.25 (2.08-8.67)	15.0 (13.0-16.9)	4.08 (3.29-5.06)	4.6 (2.9-6.3)	1.25 (0.83-1.89)^c^	3.7 (3.0-4.3)	1 [Reference]
In-hospital death rate	0.2 (0.0-0.4)	2.43 (0.49-12.04)^c^	0	NA	0.5 (0.2-0.9)	6.38 (1.69-24.05)	0	NA	0.1 (0.0-0.2)	1 [Reference]
**18-49 y**										
Hospitalization rate	566.1 (556.2-576.0)	4.98 (4.85-5.12)	744.3 (691.5-797.1)	6.56 (6.09-7.06)	423.8 (416.0-431.6)	3.73 (3.63-3.83)	135.7 (129.7-141.6)	1.19 (1.13-1.25)	113.5 (111.2-115.8)	1 [Reference]
ICU admission rate	114.3 (109.8-118.7)	5.59 (5.26-5.96)	211.6 (183.4-239.7)	10.36 (8.99-11.93)	65.0 (61.9-68.1)	3.18 (2.98-3.40)	34.0 (31.0-37.0)	1.67 (1.51-1.84)	20.4 (19.4-21.4)	1 [Reference]
In-hospital death rate	17.8 (16.0-19.5)	6.59 (5.58-7.78)	91.1 (72.7-109.6)	33.78 (26.50-43.08)	6.0 (5.1-6.9)	2.22 (1.81-2.72)	3.0 (2.1-3.9)	1.10 (0.80-1.52)^c^	2.7 (2.3-3.1)	1 [Reference]
**50-64 y**										
Hospitalization rate	1379.9 (1350.0-1409.8)	3.73 (3.63-3.83)	1725.4 (1592.7-1858.2)	4.66 (4.31-5.04)	1250.6 (1229.0-1272.1)	3.38 (3.30-3.46)	440.1 (421.4-458.8)	1.19 (1.14-1.24)	370.4 (364.4-376.3)	1 [Reference]
ICU admission rate	459.3 (442.0-476.5)	5.14 (4.89-5.40)	626.0 (546.1-706.0)	7.01 (6.10-7.10)	282.5 (272.3-292.8)	3.16 (3.01-3.32)	149.6 (138.7-160.5)	1.68 (1.55-1.81)	89.3 (86.4-92.2)	1 [Reference]
In-hospital death rate	155.5 (145.5-165.6)	6.09 (5.58-6.66)	242.1 (192.3-291.8)	9.48 (7.65-11.75)	98.7 (92.7-104.8)	3.87 (3.55-4.22)	43.5 (37.6-49.3)	1.70 (1.47-1.98)	25.5 (24.0-27.1)	1 [Reference]
**≥65 y**										
Hospitalization rate	2376.9 (2321.7-2432.1)	2.12 (2.07-2.18)	2607.6 (2402.3-2812.8)	2.33 (2.15-2.52)	2533.8 (2494.3-2573.2)	2.26 (2.22-2.30)	1009.3 (975.7-1042.8)	0.90 (0.87-0.93)	1120.4 (1109.4-1131.5)	1 [Reference]
ICU admission rate	763.2 (731.9-794.5)	3.36 (3.21-3.52)	1108.4 (974.6-1242.2)	4.88 (4.32-5.52)	698.5 (677.7-719.2)	3.08 (2.96-3.19)	476.2 (453.2-499.3)	2.10 (1.99-2.21)	227.1 (222.2-232.1)	1 [Reference]
In-hospital death rate	585.7 (558.3-613.1)	3.23 (3.07-3.41)	855.6 (738.0-973.2)	4.72 (4.11-5.43)	401.4 (385.7-417.1)	2.21 (2.11-2.32)	296.4 (278.2-314.5)	1.64 (1.53-1.75)	181.3 (176.8-185.7)	1 [Reference]

Abbreviations: ICU, intensive care unit; NA, not applicable; RR, rate ratio.

^a^ Cumulative hospitalization rates per 100 000 population were calculated using all hospitalized persons in COVID-19-Associated Hospitalization Surveillance Network with known race and ethnicity for the numerator and National Center for Health Statistics vintage 2019 bridged-race population estimates for the denominator. ICU admission and in-hospital death status were only available for sampled hospitalized patients with known race and ethnicity, complete medical record review, and a known discharge disposition; therefore, cumulative rates of ICU admission and in-hospital death per 100 000 population were calculated using weighted frequencies as the numerator and National Center for Health Statistics vintage 2019 bridged-race population estimates for the denominator.

^b^ Rates for all ages combined are age-adjusted.

^c^ RR not statistically significant.

**Figure 2.**
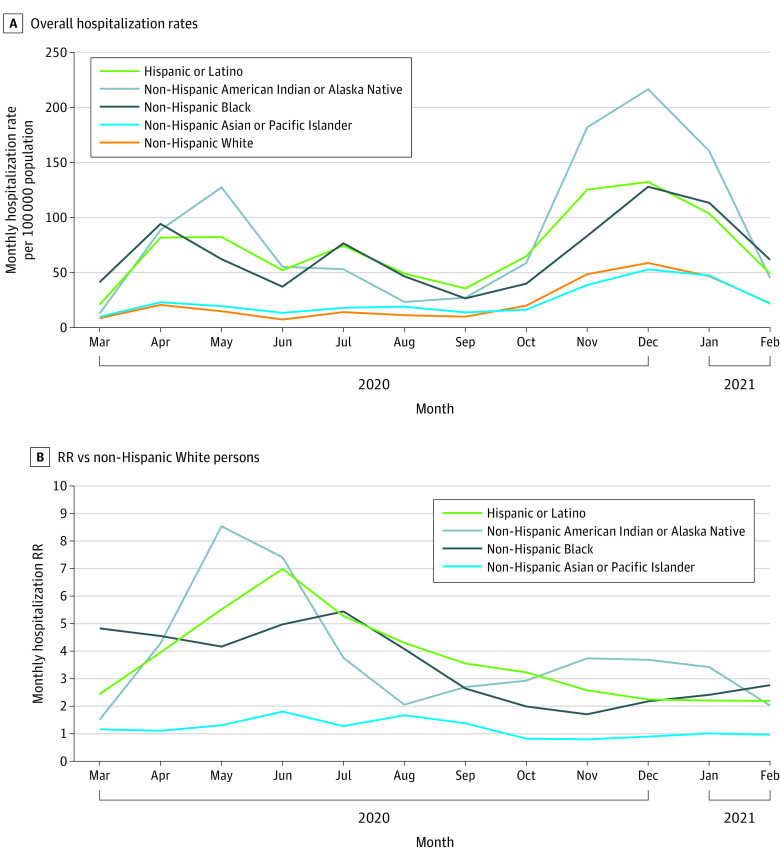
Monthly Age-Adjusted COVID-19–Associated Hospitalization Rates and Rate Ratios, by Race and Ethnicity, United States, March 1, 2020, to February 28, 2021 All rate ratios statistically significant with *P* < .01 except for rate ratios among non-Hispanic Asian or Pacific Islander persons in January 2021 and February 2021. Data are from the COVID-19-Associated Hospitalization Surveillance Network.

When examining monthly age-adjusted COVID-19–associated hospitalization rates, 3 distinct peaks were observed, the first in April to May 2020, the second in July 2020, and the third in December 2020, which had the highest hospitalization rates across all race and ethnicity groups (Figure 2A). During every month, the highest age-adjusted hospitalization rates occurred among American Indian or Alaska Native, Latino, and Black persons. For example, in May, the hospitalization rates were 127.5, 82.4, and 62.2 per 100 000 population among American Indian or Alaska Native, Latino, and Black persons, respectively, compared with 14.9 per 100 000 for White persons (Figure 2A and eTable 3 in the Supplement). Asian or Pacific Islander persons had more modestly elevated monthly hospitalization rates compared with White persons until October 2020, when this population’s rate became less than that of White persons. Trends in monthly hospitalization rates varied by age group (eFigure 1 in the Supplement).

Monthly hospitalization RRs for racial and ethnic minority groups compared with White persons peaked from May to June 2020 and then decreased but persisted over time (Figure 2B and eTable 3 in the Supplement). The highest hospitalization RR for American Indian or Alaska Native persons was 8.55 (95% CI, 7.50-9.74) in May 2020; for Asian or Pacific Islander persons, 1.81 (95% CI, 1.61-2.03) in June 2020; for Black persons, 5.44 (95% CI, 5.19-5.71) in July 2020; and for Latino persons, 6.99 (95% CI, 6.53-7.49) in June 2020. By February 2021, RRs were 2.03 (95% CI, 1.64-2.50), 0.97 (95% CI, 0.89-1.06), 2.77 (95% CI, 2.64-2.90), and 2.20 (95% CI, 2.07-2.23) for American Indian or Alaska Native persons, Asian or Pacific Islander persons, Black persons, and Latino persons, respectively, compared with White persons. Notably, after an initial decline, monthly RRs for Black persons increased for 3 consecutive months during December 2020 to February 2021.

**Figure 3.**
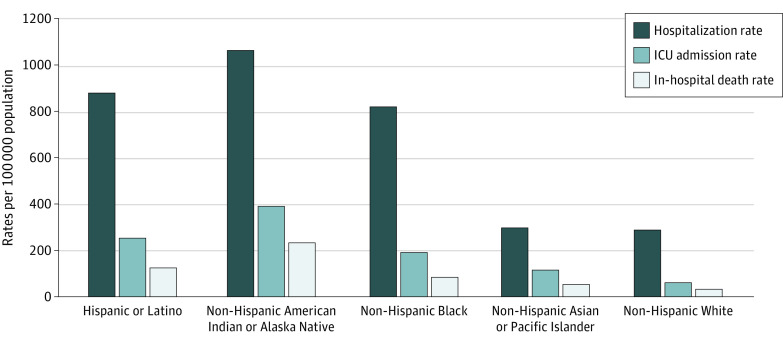
Cumulative Age-Adjusted Hospitalization, Intensive Care Unit (ICU) Admission, and In-Hospital Death Rates by Race and Ethnicity, United States, March 1, 2020 to February 28, 2021 Cumulative hospitalization rates per 100 000 population were calculated using all hospitalized persons in the COVID-19–Associated Hospitalization Surveillance Network with known race and ethnicity for the numerator and National Center for Health Statistics vintage 2019 bridged-race population estimates for the denominator. ICU admission and in-hospital death status were only available for sampled hospitalized patients with known race and ethnicity, complete medical record review, and a discharge disposition; therefore, cumulative rates of ICU admission and in-hospital death per 100 000 population were calculated using weighted frequencies as the numerator and National Center for Health Statistics vintage 2019 bridged-race population estimates for the denominator.

Cumulative age-adjusted ICU admission and death rates were highest among American Indian or Alaska Native persons, followed by Latino and Black persons (Table 1 and Figure 3). Compared with White persons, cumulative age-adjusted ICU admission and death RRs were as follows: American Indian or Alaska Native (ICU admission: RR, 6.49; 95% CI, 6.01-7.01; death: RR, 7.19; 95% CI, 6.47-7.99); Asian or Pacific Islander (ICU admission: RR, 1.91; 95% CI, 1.83-1.98; death: RR, 1.64; 95% CI 1.55-1.74); Black (ICU admission: RR, 3.17; 95% CI, 3.09-3.26; death: RR, 2.58; 95% CI, 2.48-2.69); and Latino (ICU admission: RR, 4.20; 95% CI, 4.08-4.33; death: RR, 3.85; 95% CI, 3.68-4.01). For each adult age group, American Indian or Alaska Native, followed by Latino and then Black persons, had the highest cumulative rates of ICU admission and death; the largest rate disparities occurred in these racial and ethnic groups among adults aged 18 to 49 years (Table 1).

Cumulative age-adjusted rates of COVID-19–associated hospitalization, ICU admission, and death by race and ethnicity varied by site (eFigure 2 in the Supplement). Hospitalization rates were highest for Latino persons followed by Black persons in 7 sites (California, Colorado, Georgia, Maryland, Ohio, Oregon, and Tennessee); rates were highest for Black persons followed by Latino persons in 3 sites (Connecticut, Michigan, and New York); and rates were highest for American Indian or Alaska Native persons in 3 sites (New Mexico, Minnesota, and Utah).

### Frequency of Clinical Interventions and Outcomes Among a Weighted Sample of Hospitalized Patients

Among 25 281 sampled hospitalized patients of all ages, compared with White patients, Asian or Pacific Islander and Latino patients had significantly higher percentages with ICU admission, IMV use, and vasopressor use; Black patients had a significantly higher percentage with KRT (Table 2). Median LOS was similar among race and ethnicity groups (range, 4.5-5.3 days). The frequency of clinical interventions and outcomes by race and ethnicity among hospitalized patients varied by age group (Table 2).

**Table 2.** Weighted Prevalence of Clinical Interventions and Outcomes Among a Sample of 25 281 Hospitalized Patients by Race and Ethnicity and Age Group, United States, March 1, 2020, to February 28, 2021

Intervention or outcome	Weighted % (95% CI)						
	Hispanic or Latino	Non-Hispanic					
		American Indian or Alaska Native	Black		Asian or Pacific Islander	White	
**All ages**							
ICU admission	27.2 (25.0-29.4)	29.2 (21.6-36.7)	22.9 (20.9-24.9)	29.7 (24.4-35.1)			21.1 (19.7-22.5)
IMV	17.0 (15.0-19.1)	16.3 (10.5-22.1)	12.9 (11.3-14.5)	17.8 (12.6-23.1)			10.7 (9.6-11.8)
Vasopressor use	15.3 (13.3-17.2)	15.9 (10.1-21.6)	12.5 (10.9-14.1)	17.3 (12.1-22.5)			10.2 (9.2-11.2)
Kidney replacement therapy	5.3 (4.2-6.4)	7.6 (2.4-12.7)	9.1 (7.6-10.6)	3.6 (1.9-5.3)			4.0 (3.3-4.8)
In-hospital death	10.8 (8.8-12.7)	16.0 (9.6-22.4)	9.2 (7.8-10.5)	13.1 (8.2-18.0)			12.1 (10.9-13.3)
LOS, median (IQR), d	4.5 (4.2-4.7)	5.3 (3.9-6.6)	4.8 (4.6-5.1)	4.8 (4.1-5.4)			4.9 (4.7-5.0)
**<18 y**							
ICU admission	23.3 (20.4-26.1)	32.9 (14.2-51.7)	33.5 (30.0-37.1)		22.6 (15.1-30.1)	25.2 (21.4-28.9)	
IMV	5.3 (3.8-6.8)	12.4 (0.0-25.5)	5.4 (3.7-7.1)		2.5 (0.0-5.2)	6.3 (4.2-8.4)	
Vasopressor use	8.2 (6.3-10.1)	4.2 (0.0-12.1)	8.5 (6.4-10.6)		5.7 (1.6-9.9)	5.7 (3.7-7.7)	
Kidney replacement therapy	0.6 (0.1-1.1)	0	0.6 (0.0-1.2)		0.8 (0.0-2.4)	0.4 (0.0-0.9)	
In-hospital death	0.4 (0.0-0.8)	0	1.2 (0.4-2.0)		0	0.6 (0.0-1.2)	
LOS, median (IQR), d	2.1 (1.9-2.3)	3.0 (1.7-4.2)	2.6 (2.4-2.8)		1.8 (1.5-2.1)	2.0 (1.8-2.3)	
**18-49 y**							
ICU admission	19.5 (17.1-21.9)	29.0 (20.3-37.7)	15.7 (12.8-18.5)		18.2 (12.8-23.7)	18.4 (15.3-21.5)	
IMV	10.9 (8.9-13.0)	14.8 (7.7-22.0)	7.9 (5.7-10.1)		6.6 (3.6-9.6)	8.6 (6.5-10.6)	
Vasopressor use	9.1 (7.2-10.9)	14.9 (7.9-21.8)	7.6 (5.3-9.8)		6.3 (3.2-9.3)	6.9 (5.0-8.8)	
Kidney replacement therapy	3.4 (2.1-4.8)	8.0 (2.4-13.5)	5.6 (3.7-7.6)		3.2 (0.8-5.6)	2.4 (1.1-3.6)	
In-hospital death	3.0 (1.9-4.2)	12.5 (5.3-19.6)	1.4 (0.7-2.2)		1.6 (0.2-2.9)	2.4 (1.2-3.7)	
LOS, median (IQR), d	3.5 (3.3-3.8)	4.2 (3.0-5.5)	3.4 (3.0-3.7)		3.4 (2.7-4.0)	3.3 (3.0-3.6)	
**50-64 y**							
ICU admission	32.1 (28.1-36.0)	36.1 (23.9-48.3)	22.6 (19.6-25.5)		30.5 (23.3-37.6)	24.3 (21.6-26.9)	
IMV	20.0 (16.6-23.5)	20.0 (11.6-28.4)	13.7 (11.2-16.1)		15.3 (9.9-20.8)	11.9 (9.9-13.9)	
Vasopressor use	19.3 (15.8-22.8)	18.7 (10.7-26.6)	12.7 (10.4-15.1)		14.9 (9.4-20.3)	11.7 (9.7-13.7)	
Kidney replacement therapy	7.6 (5.3-9.9)	9.5 (0.0-19.9)	9.3 (7.1-11.4)		6.9 (2.0-11.9)	4.9 (3.6-6.2)	
In-hospital death	10.8 (7.9-13.8)	13.4 (6.5-20.3)	7.7 (5.8-9.5)		8.8 (3.8-13.9)	7.0 (5.2-8.8)	
LOS, median (IQR), d	5.4 (4.9-5.9)	7.1 (5.2-9.1)	4.8 (4.4-5.1)		4.6 (3.6-5.5)	4.5 (4.2-4.7)	
**≥65 y**							
ICU admission	35.7 (29.4-41.9)	25.4 (10.8-40.0)	28.4 (24.2-32.5)		35.5 (25.6-45.4)	20.6 (18.7-22.6)	
IMV	26.5 (20.2-32.8)	14.8 (3.2-26.5)	16.1 (12.9-19.2)		25.7 (15.7-35.7)	10.9 (9.4-12.5)	
Vasopressor use	22.9 (17.1-28.7)	14.9 (3.3-26.6)	16.5 (13.2-19.9)		24.9 (14.9-34.8)	10.7 (9.2-12.2)	
Kidney replacement therapy	6.8 (4.2-9.5)	6.1 (0.0-15.5)	11.8 (8.7-15.0)		2.2 (0.1-4.2)	4.1 (3.0-5.2)	
In-hospital death	27.2 (21.0-33.5)	20.6 (6.5-34.7)	15.9 (12.8-18.9)		22.1 (12.7-31.4)	16.2 (14.4-17.9)	
LOS, median (IQR), d	6.2 (5.1-7.3)	5.5 (3.1-7.8)	6.2 (5.5-6.8)		7.4 (4.8-10.0)	5.6 (5.3-6.0)	

Abbreviations: ICU, intensive care unit; IMV, invasive mechanical ventilation; LOS, length of hospital stay.

## Discussion

Within a large, multisite, US population–based surveillance network with robust methods for case ascertainment and highly complete information on race and ethnicity, we identified racial and ethnic disparities in rates of severe COVID-19 during the first year of the COVID-19 pandemic. American Indian or Alaska Native, Latino, Black, and Asian or Pacific Islander persons were significantly more likely to be hospitalized, receive ICU care, or die with COVID-19–associated illness compared with White persons. These disparities were present across all age groups and persisted during the entire 12-month surveillance period. After peaking in May through July 2020, disparities in monthly hospitalization rates among racial and ethnic minority groups appeared to decrease; however, there was a concerning increase in hospitalization disparities among Black persons in December 2020 to February 2021. The observed trends were in part due to increasing hospitalization rates among White persons over time rather than substantial declines in hospitalization rates among other racial and ethnic minority groups. This analysis is among the first to offer a detailed longitudinal picture of ongoing racial and ethnic disparities related to severe COVID-19 in the United States.

Our findings build on previous COVID-NET studies^3,4,5^ and other assessments that found that Black populations are disproportionately hospitalized with COVID-19. Within regional integrated health care systems in California, 2 studies demonstrated that Black persons had 1.5 to 2.7 times higher risks of hospitalization compared with White persons, even after adjusting for various factors.^7,13^ Other single-site studies found that Black persons were 1.7 to 2.0 times more likely to be hospitalized with COVID-19 than White or other racial and ethnic minority groups.^6,14,15^ Our analysis also demonstrated greater rates of COVID-19–associated hospitalization among American Indian or Alaska Native, Asian or Pacific Islander, and Latino populations, groups for whom data are limited and conflicting. Some studies have found higher risk of hospitalization among Asian or Pacific Islander and Latino persons,^7,8,16,17^ while others have found no difference in risk compared with White persons.^13,18^

We identified increased age-adjusted rates of ICU admission and in-hospital death among American Indian or Alaska Native, Latino, Black, and Asian or Pacific Islander populations in comparison with White populations. Multiple studies have shown that once hospitalized, Black patients do not have increased risk of ICU admission or death compared with White patients,^4,6,7,8,14,15,18,19,20,21^ even after adjusting for factors such as sex, medical comorbidities, and socioeconomic factors. Studies among Asian or Pacific Islander and Latino persons have shown varied results; while some found no increased risk of severe outcomes in these groups,^7,8,22^ others found a higher risk of death^18,23^ compared with White persons. However, few of these studies have used population-based data to examine the association of race and ethnicity with rates of severe COVID-19–associated disease while taking the underlying population into account. Additionally, disparities in access to health care or potential biases related to hospital admission were not considered in many studies, affecting the estimation of COVID-19–associated complications and death.

There is a dearth of information regarding rates of severe COVID-19 disease among American Indian or Alaska Native populations who account for 0.8% of the US population^24^; our findings indicate that these populations shoulder a disproportionate burden of severe COVID-19–associated disease, with the highest overall rates of hospitalization, ICU admission, and death. We also found that rates of severe COVID-19 among American Indian or Alaska Native persons varied substantially by geography and were largely related to 3 surveillance sites with larger American Indian or Alaska Native populations. While data on risk of COVID-19–associated hospitalization among American Indian or Alaska Native persons are limited, 1 study found that COVID-19 incidence among these populations was 3.5-fold higher than that among White persons^25^; other studies have found COVID-19–associated mortality rates to be 1.8 to 2.2 times greater than those of White persons.^26,27^ Similar disparities among American Indian or Alaska Native populations were also observed during the 2009 influenza A H1N1 pandemic.^28^

This analysis adds to limited information published on severe COVID-19 disease in Asian or Pacific Islander populations. While Asian or Pacific Islander persons had modestly higher rates of hospitalization, ICU admission, and in-hospital death compared with White persons, the aggregation of multiple Asian or Pacific Islander populations within 1 category, which was necessary to produce rates based on NCHS data, may have obscured disparities. Other evaluations focused specifically on Pacific Island persons have demonstrated much higher rates of hospitalization and death; reports of increased COVID-19 incidence in Pacific Islander persons from disparate US jurisdictions support these findings.^23,27,29,30^ These data suggest that disaggregation of groups within the Asian or Pacific Islander category might uncover complex disparities.

The cause of higher hospitalization, ICU admission, and in-hospital death rates across racial and ethnic minority populations is likely multifactorial. Higher prevalence of hypertension, obesity, and chronic kidney disease among Black persons as well as higher prevalence of diabetes among American Indian or Alaska Native persons may play a role in the increased rates of severe COVID-19 disease in these populations.^4,5,31^ Community-level exposure to and incidence of COVID-19 is also likely a large driver of disparities in severe COVID-19 disease.^32,33^ Importantly, members of racial and ethnic minority groups face inequity due to structural racism, with its many downstream consequences on overall health, including poor access to health care and economic instability.^34,35^ Poverty, unstable housing, lack of transportation, and poor access to quality education, among other social determinants of health, are more common in American Indian or Alaska Native, Asian or Pacific Islander, Latino, and Black populations.^36,37,38^ Additionally, these populations are more likely to work in essential industries and live in larger, multigenerational households, increasing the risk of exposure to COVID-19.^38,39,40,41^ Other barriers to health care, including lack of health insurance, a primary language other than English, low health literacy, and differing levels of acculturation, are also observed more frequently in American Indian or Alaska Native, Asian or Pacific Islander, Latino, and Black populations.^25,42^ Together, these factors may intensify disparities in health outcomes, including the observed rates of severe COVID-19 disease.

### Limitations

Several limitations should be considered. COVID-NET relies on clinician-driven or facility-based testing practices to identify cases; rates are likely underestimated, as some patients hospitalized with COVID-19 may not have been tested. While rates are age-adjusted, we were unable to adjust for other important factors, including underlying medical conditions and socioeconomic indicators, as these data were not available for the COVID-NET surveillance population. Future analyses that link COVID-NET data to other sources of population-level health data will be important in understanding the impact of these factors on COVID-19–associated outcomes. Due to the relatively low rates of COVID-19–associated hospitalizations among children, estimates by race and ethnicity within this age group were subject to variability. Although this is among the few analyses to include population-based data from geographically diverse sites, COVID-NET represents approximately 10% of the US population and findings may not be generalizable to the entire country. While it is reassuring that the COVID-NET racial and ethnic makeup is similar to that of the US population (COVID-NET population: Latino, 14.1%; American Indian or Alaska Native, 0.7%; Asian or Pacific Islander, 8.9%; Black, 17.9%; White, 58.5%; U.S population: Latino, 18.5%; American Indian or Alaska Native, 0.8%; Asian or Pacific Islander, 6.3%; Black, 13.2%; White, 61.2%), findings were likely affected by differences in racial and ethnic distributions and COVID-19 disease incidence across sites. Race and ethnicity classifications were limited to those available through the NCHS, and we could not evaluate specific groups that may be disproportionately affected by COVID-19, such as Pacific Islander persons, or people of more than 1 race and ethnicity. Finally, 5.9% of hospitalizations had missing or unknown ethnicity, which were presumed to be non-Latino; therefore, rates may be underreported for Latino persons.

## Conclusions

This longitudinal analysis found that racial and ethnic minority groups have experienced severe COVID-19 outcomes disproportionately in the United States during the first year of the COVID-19 pandemic. Further work is needed to understand the complex relationship between race and ethnicity and COVID-19–associated outcomes. In addition, an emphasis on studying how socioeconomic inequities, structural racism, and cultural differences can result in immediate and long-term barriers to adequate health care for these populations may lead to successful interventions that improve health. Nonetheless, because of the current disproportionately high burden of severe COVID-19 among racial and ethnic minority groups, equitable access to preventive measures, such as vaccination, and treatments should be urgently optimized among these groups.

## Notes

Supplement. **eTable 1. **Demographic Characteristics of All vs Sampled Hospitalized Patients, COVID-NET, United States, March 1, 2020–February 28, 2021 **eTable 2. **Weighted Prevalence of Select Underlying Medical Conditions in a Sample of 25 281 Hospitalized Patients by Race and Ethnicity and Age Group, COVID-NET, United States, March 1, 2020–February 28, 2021 **eTable 3. **Monthly Age-Adjusted COVID-19–Associated Hospitalization Rates (per 100 000 Population) and Rate Ratios by Race and Ethnicity, COVID-NET, United States, March 1, 2020–February 28, 2021 **eFigure 1. **Monthly COVID-19–Associated Hospitalization Rates (per 100 000 Population) by Race and Ethnicity and Age Group, COVID-NET, United States, March 1, 2020–February 28, 2021 **eFigure 2. **Age-Adjusted Hospitalization, ICU Admission, and In-Hospital Mortality Rates by Race and Ethnicity and State, COVID-NET, March 1, 2020–February 28, 2021
