# Supplementary material for: Racial and Ethnic Disparities in Rates of COVID-19–Associated Hospitalization, Intensive Care Unit Admission, and In-Hospital Death in the United States From March 2020 to February 2021
Source: JAMA Netw Open. 2021 Oct 21;4(10):e2130479. doi: 10.1001/jamanetworkopen.2021.30479 (PMC8531997; doi:10.1001/jamanetworkopen.2021.30479)
Supplement: Supplement. — eTable 1. Demographic Characteristics of All vs Sampled Hospitalized Patients, COVID-NET, United States, March 1, 2020–February 28, 2021 eTable 2. Weighted Prevalence of Select Underlying Medical Conditions in a Sample of 25 281 Hospitalized Patients by Race and Ethnicity and Age Group, COVID-NET, United States, March 1, 2020–February 28, 2021 eTable 3. Monthly Age-Adjusted COVID-19–Associated Hospitalization Rates (per 100 000 Population) and Rate Ratios by Race and Ethnicity, COVID-NET, United States, March 1, 2020–February 28, 2021 eFigure 1. Monthly COVID-19–Associated Hospitalization Rates (per 100 000 Population) by Race and Ethnicity and Age Group, COVID-NET, United States, March 1, 2020–February 28, 2021 eFigure 2. Age-Adjusted Hospitalization, ICU Admission, and In-Hospital Mortality Rates by Race and Ethnicity and State, COVID-NET, March 1, 2020–February 28, 2021 [file jamanetwopen-e2130479-s001.pdf]

Supplemental Online Content

Acosta AM, Garg S, Pham H, et al. Racial and ethnic disparities in rates of COVID-19–associated hospitalization, intensive care unit admission, and in-hospital death in the United States from March 2020 to February 2021. *JAMA Netw Open.* 2021;4(10):e2130479. doi:10.1001/jamanetworkopen.2021.30479

eTable 1. Demographic Characteristics of All vs Sampled Hospitalized Patients, COVID-NET, United States, March 1, 2020–February 28, 2021

eTable 2. Weighted Prevalence of Select Underlying Medical Conditions in a Sample of 25 281 Hospitalized Patients by Race and Ethnicity and Age Group, COVID-NET, United States, March 1, 2020–February 28, 2021

eTable 3. Monthly Age-Adjusted COVID-19–Associated Hospitalization Rates (per 100 000 Population) and Rate Ratios by Race and Ethnicity, COVID-NET, United States, March 1, 2020–February 28, 2021

eFigure 1. Monthly COVID-19–Associated Hospitalization Rates (per 100 000 Population) by Race and Ethnicity and Age Group, COVID-NET, United States, March 1, 2020–February 28, 2021

eFigure 2. Age-Adjusted Hospitalization, ICU Admission, and In-Hospital Mortality Rates by Race and Ethnicity and State, COVID-NET, March 1, 2020–February 28, 2021

This supplemental material has been provided by the authors to give readers additional information about their work.

© 2021 Acosta AM et al. *JAMA Network Open.*

eTable 1. Demographic Characteristics of All vs Sampled Hospitalized Patients, COVID-NET, United States, March 1, 2020–February 28, 2021

Characteristic	All Hospitalized Patients[a]		Sampled Hospitalized Patients[b]	
	Unweighted n	Unweighted %	Unweighted n	Weighted %
Total	143,342	100	25,281	100
**Age category**				
0–17 years	2,202	1.5	2,188	1.5
18–49 years	35,719	24.9	8,429	24.9
50–64 years	38,860	27.1	6,484	27.2
≥65 years	66,561	46.4	8,180	46.4
**Race and Ethnicity**				
Hispanic or Latino	28,762	20.1	6,134	19.7
Non-Hispanic American Indian or Alaska Native	2,056	1.4	513	1.7
Non-Hispanic Black	40,806	28.5	5,728	27.8
Non-Hispanic Asian or Pacific Islander	7,737	5.4	2,005	6.7
Non-Hispanic White	63,981	44.6	10,901	44.0
**Sex**				
Male	72,159	50.3	12,768	50.2
Female	71,181	49.7	12,513	49.8
**Surveillance Site**				
California	11,011	7.7	1,084	7.9
Colorado	11,366	7.9	1,725	8.1
Connecticut	7,131	5.0	1,181	4.8
Georgia	24,200	16.9	1,537	16.5
Iowa	796	0.6	191	0.5
Maryland	32,495	22.7	2,088	22.7
Michigan	4,467	3.1	808	3.1
Minnesota	13,690	9.6	6,681	9.8
New Mexico	6,143	4.3	887	4.4
New York	10,047	7.0	1085	7.0
Ohio	4,066	2.8	938	2.8
Oregon	3,030	2.1	796	2.2
Tennessee	10,176	7.1	2,355	7.0
Utah	4,724	3.3	3,925	3.2

[a] Includes the hospitalizations included in the analysis (n=143,342)
[b] Includes sampled patients with completed chart review and a discharge disposition (n=25,281)

© 2021 Acosta AM et al. *JAMA Network Open.*

eTable 2. Weighted Prevalence of Select Underlying Medical Conditions in a Sample of 25 281 Hospitalized Patients by Race and Ethnicity and Age Group, COVID-NET, United States, March 1, 2020–February 28, 2021

Condition	Age group	Hispanic or Latino (%)	Non-Hispanic American Indian or Alaska Native (%)	Non-Hispanic Black (%)	Non-Hispanic Asian or Pacific Islander (%)	Non-Hispanic White (%)
Any underlying condition	**All ages**	**82.1**	**90.0**	**94.4**	**88.9**	**94.4**
	0-17 years	54.8	58.4	60.6	46.8	44.1
	18-49 years	78.8	88.6	91.3	86.0	86.0
	50-64 years	81.7	95.0	94.5	83.9	93.0
	65+ years	92.8	88.4	98.0	94.5	97.4
Hypertension	**All ages**	**37.7**	**52.3**	**67.6**	**54.6**	**63.9**
	0-17 years	1.8	0.0	2.3	1.7	0.8
	18-49 years	15.9	20.7	37.1	17.9	28.0
	50-64 years	48.6	67.6	73.3	54.2	56.4
	65+ years	72.6	66.4	87.2	78.5	75.6
Obesity	**All ages**	**51.2**	**47.6**	**55.1**	**31.7**	**45.0**
	0-17 years	43.9	66.9	33.3	37.0	20.4
	18-49 years	60.4	69.4	66.7	57.6	63.9
	50-64 years	50.7	64.6	64.8	34.3	59.6
	65+ years	36.8	21.3	40.8	18.0	36.2
Chronic metabolic disease	**All ages**	**38.6**	**55.0**	**44.5**	**47.1**	**44.2**
	0-17 years	4.3	4.1	5.6	5.0	5.6
	18-49 years	23.0	36.1	26.6	25.4	25.3
	50-64 years	49.4	61.7	46.8	45.1	44.5
	65+ years	60.5	65.6	57.1	62.6	48.9
Diabetes mellitus	**All ages**	**33.6**	**48.3**	**41.1**	**42.2**	**32.3**
	0-17 years	3.0	4.1	4.7	0.8	2.9
	18-49 years	19.2	32.2	23.9	23.7	17.1
	50-64 years	45.0	58.4	42.7	37.7	34.4
	65+ years	52.0	54.4	53.6	57.0	35.3
Chronic lung disease	**All ages**	**16.5**	**19.8**	**28.5**	**19.6**	**33.6**

© 2021 Acosta AM et al. *JAMA Network Open.*

	0-17 years	13.8	25.5	21.7	17.5	14.4
	18-49 years	14.2	18.1	24.6	13.8	25.0
	50-64 years	17.3	28.8	29.5	18.4	34.3
	65+ years	20.5	15.0	30.6	23.8	35.6
Asthma	**All ages**	**10.2**	**11.6**	**15.5**	**9.9**	**10.7**
	0-17 years	10.2	25.5	18.9	12.5	10.9
	18-49 years	10.4	12.5	18.7	9.0	15.8
	50-64 years	10.3	18.7	14.8	8.1	14.6
	65+ years	9.9	5.9	13.7	11.3	8.1
Chronic Obstructive Pulmonary Disease	**All ages**	**2.2**	**3.0**	**8.0**	**3.0**	**16.5**
	0-17 years	0.1	0.0	0.0	0.0	0.0
	18-49 years	0.1	0.1	0.4	0.0	2.3
	50-64 years	0.9	3.8	8.1	1.6	12.6
	65+ years	8.5	4.6	13.7	5.6	21.2
Cardiovascular disease	**All ages**	**16.2**	**27.5**	**36.8**	**28.5**	**46.7**
	0-17 years	5.7	0.0	4.7	6.7	4.1
	18-49 years	5.9	6.2	13.8	5.4	11.1
	50-64 years	15.4	30.5	37.2	18.8	33.5
	65+ years	39.8	41.6	54.2	48.2	60.1
Coronary artery disease	**All ages**	**4.9**	**12.2**	**10.5**	**9.6**	**19.1**
	0-17 years	0.0	0.0	0.2	0.0	0.0
	18-49 years	0.6	0.8	1.7	0.3	1.4
	50-64 years	5.6	9.4	10.2	8.3	11.4
	65+ years	13.3	22.5	17.4	16.3	26.1
Heart failure	**All ages**	**5.0**	**13.1**	**15.2**	**9.7**	**14.2**
	0-17 years	0.1	0.0	0.2	0.0	0.2
	18-49 years	2.4	1.6	7.1	0.6	2.1
	50-64 years	3.3	3.2	15.1	5.4	9.7
	65+ years	13.1	28.1	21.7	17.8	18.8
Neurologic disease	**All ages**	**9.7**	**18.5**	**20.4**	**14.4**	**26.9**
	0-17 years	13.2	12.5	13.3	12.5	13.3
	18-49 years	3.7	9.2	10.3	6.3	14.7
	50-64 years	7.1	13.6	14.8	6.8	18.6

© 2021 Acosta AM et al. *JAMA Network Open.*

	65+ years	24.9	28.6	32.7	23.4	32.9
Renal disease	**All ages**	**9.5**	**12.6**	**21.3**	**17.7**	**17.6**
	0-17 years	0.7	0.0	1.8	1.7	1.0
	18-49 years	4.3	4.2	8.7	7.4	4.7
	50-64 years	11.2	13.5	20.7	10.8	12.4
	65+ years	19.0	18.5	31.6	27.9	22.6
Immunosuppressive condition	**All ages**	**6.5**	**6.4**	**11.6**	**7.8**	**11.5**
	0-17 years	5.1	4.5	3.6	4.2	5.0
	18-49 years	3.9	2.5	8.4	3.7	7.7
	50-64 years	9.4	4.8	13.7	4.9	12.3
	65+ years	8.2	10.3	12.4	12.0	12.1
Gastrointestinal or liver disease	**All ages**	**3.5**	**5.6**	**4.9**	**5.9**	**6.2**
	0-17 years	1.5	0.0	0.8	1.7	1.0
	18-49 years	2.3	13.1	3.0	3.5	7.2
	50-64 years	4.7	5.5	6.3	3.9	7.6
	65+ years	4.6	0.4	5.2	8.6	5.5
Hematologic condition	**All ages**	**1.5**	**4.3**	**2.9**	**3.3**	**3.5**
	0-17 years	2.3	0.0	12.1	1.7	1.9
	18-49 years	1.0	2.3	4.2	2.2	1.5
	50-64 years	1.7	0.9	1.2	1.3	3.1
	65+ years	2.1	7.9	3.1	5.0	4.2
Rheumatologic or autoimmune disease	**All ages**	**1.8**	**4.5**	**2.9**	**1.8**	**4.0**
	0-17 years	0.5	0.0	0.3	2.6	0.2
	18-49 years	1.0	2.1	2.8	1.8	2.0
	50-64 years	2.8	2.3	3.2	2.4	4.3
	65+ years	2.2	7.8	3.0	1.5	4.3

[a] Data are collected on underlying medical conditions within the major categories of cardiovascular, chronic metabolic, chronic lung, hematologic, immunosuppressive, neurologic, rheumatologic, and renal disease, in addition to specific conditions such as diabetes mellitus type 1 or 2, hypertension, and obesity (defined as a calculated body mass index ≥30 kg/m^2).

© 2021 Acosta AM et al. *JAMA Network Open.*

eTable 3. Monthly Age-Adjusted COVID-19–Associated Hospitalization Rates (per 100 000 Population) and Rate Ratios by Race and Ethnicity, COVID-NET, United States, March 1, 2020–February 28, 2021

	Hispanic or Latino		Non-Hispanic American Indian or Alaska Native		Non-Hispanic Black		Non-Hispanic Asian or Pacific Islander		Non-Hispanic White	
	Rate (95% CI) per 100,000 population	RR (95% CI)	Rate (95% CI) per 100,000 population	RR (95% CI)	Rate (95% CI) per 100,000 population	RR (95% CI)	Rate (95% CI) per 100,000 population	RR (95% CI)	Rate (95% CI) per 100,000 population	RR (95% CI)
**Mar**	20.9 (19.2-22.5)	2.44 (2.23 - 2.68)	12.9 (7.8-18.1)	1.51 (1.02 - 2.25)	41.3 (39.5-43.1)	4.83 (4.53 - 5.14)	10.0 (8.8-11.2)	1.17 (1.02 - 1.33)	8.6 (8.2-8.9)	1 [reference]
**Apr**	81.8 (78.6-85.1)	3.95 (3.76 - 4.15)	88.7 (75.2-102.2)	4.29 (3.67 - 5.00)	94.2 (91.4-97.0)	4.55 (4.37 - 4.74)	23.0 (21.1-24.9)	1.11 (1.02 - 1.21)	20.7 (20.1-21.3)	1 [reference]
**May**	82.4 (79.2-85.6)	5.52 (5.24 - 5.81)	127.5 (111.4-143.6)	8.55 (7.50 - 9.74)	62.2 (60.0-64.5)	4.17 (3.97 - 4.38)	19.5 (17.8-21.2)	1.31 (1.19 - 1.43)	14.9 (14.4-15.4)	1 [reference]
**Jun**	52.2 (49.7-54.7)	6.99 (6.53 - 7.49)	55.3 (44.8-65.8)	7.40 (6.09 - 9.01)	37.2 (35.5-38.9)	4.98 (4.66 - 5.32)	13.5 (12.1-14.9)	1.81 (1.61 - 2.03)	7.5 (7.1-7.8)	1 [reference]
**Jul**	74.4 (71.3-77.5)	5.28 (5.00 - 5.58)	53.0 (42.8-63.3)	3.76 (3.09 - 4.58)	76.8 (74.3-79.2)	5.45 (5.19 - 5.71)	18.1 (16.5-19.7)	1.28 (1.17 - 1.41)	14.1 (13.6-14.6)	1 [reference]
**Aug**	49.0 (46.5-51.5)	4.30 (4.03 - 4.59)	23.5 (16.5-30.4)	2.06 (1.53 - 2.77)	46.5 (44.6-48.4)	4.08 (3.85 - 4.32)	19.1 (17.4-20.8)	1.67 (1.52 - 1.84)	11.4 (10.9-11.8)	1 [reference]
**Sep**	35.7 (33.5-37.8)	3.55 (3.29 - 3.82)	27.2 (19.9-34.5)	2.70 (2.06 - 3.55)	26.6 (25.2-28.1)	2.65 (2.47 - 2.84)	13.8 (12.4-15.3)	1.38 (1.23 - 1.54)	10.1 (9.6-10.5)	1 [reference]
**Oct**	64.8 (61.8-67.8)	3.23 (3.06 - 3.41)	58.8 (47.8-69.7)	2.93 (2.43 - 3.54)	39.9 (38.1-41.7)	1.99 (1.89 - 2.10)	16.4 (14.9-18.0)	0.82 (0.74 - 0.90)	20.0 (19.4-20.6)	1 [reference]

© 2021 Acosta AM et al. *JAMA Network Open.*

**Nov**	125.4 (121.1-129.6)	2.58 (2.48 - 2.68)	181.7 (162.3-201.1)	3.74 (3.35 - 4.17)	83.1 (80.5-85.7)	1.71 (1.65 - 1.77)	38.7 (36.3-41.2)	0.80 (0.75 - 0.85)	48.6 (47.7-49.5)	1 [reference]
**Dec**	132.2 (127.8-136.6)	2.25 (2.17 - 2.34)	216.4 (195.1-237.8)	3.69 (3.34 - 4.07)	127.9 (124.7-131.2)	2.18 (2.11 - 2.25)	52.9 (50.1-55.7)	0.90 (0.85 - 0.95)	58.7 (57.7-59.7)	1 [reference]
**Jan**	103.6 (99.7-107.4)	2.21 (2.12 - 2.30)	160.5 (142.1-178.9)	3.42 (3.05 - 3.84)	113.3 (110.3-116.3)	2.42 (2.34 - 2.50)	47.5 (44.8-50.2)	1.01 (0.95 - 1.08)	46.9 (46.0-47.8)	1 [reference]
**Feb**	49.1 (46.4-51.7)	2.20 (2.07 - 2.33)	45.2 (35.7-54.7)	2.03 (1.64 - 2.50)	61.8 (59.6-64.0)	2.77 (2.64 - 2.90)	21.7 (19.9-23.5)	0.97 (0.89 - 1.06)	22.3 (21.7-23.0)	1 [reference]

[a] Monthly hospitalization rates per 100,000 population were calculated using all hospitalized persons in COVID-NET with known race and ethnicity for each month for the numerator and NCHS vintage 2019 bridged-race population estimates for the denominator, and adjusting for age.

© 2021 Acosta AM et al. *JAMA Network Open.*

eFigure 1. Monthly COVID-19–Associated Hospitalization Rates (per 100 000 Population) by Race and Ethnicity and Age Group, COVID-NET, United States, March 1, 2020–February 28, 2021

A, Children aged <18 years

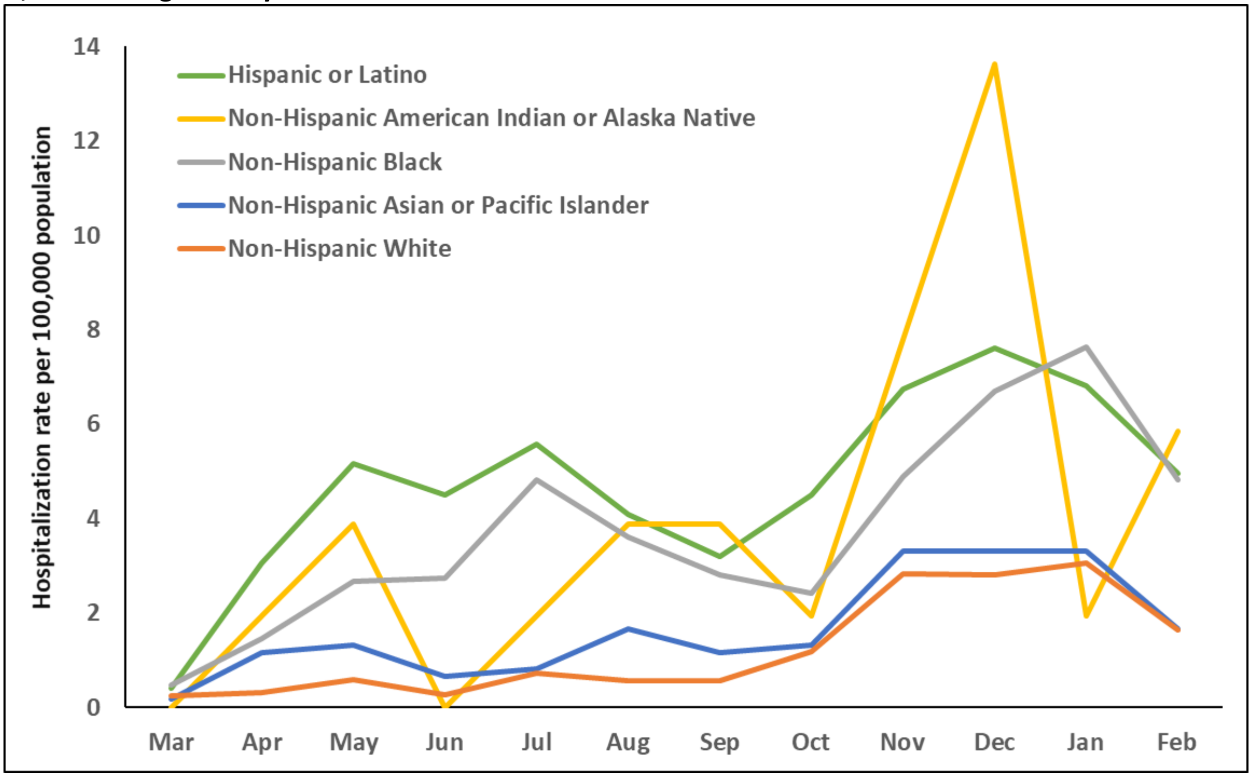

B, Adults aged 18-49 years

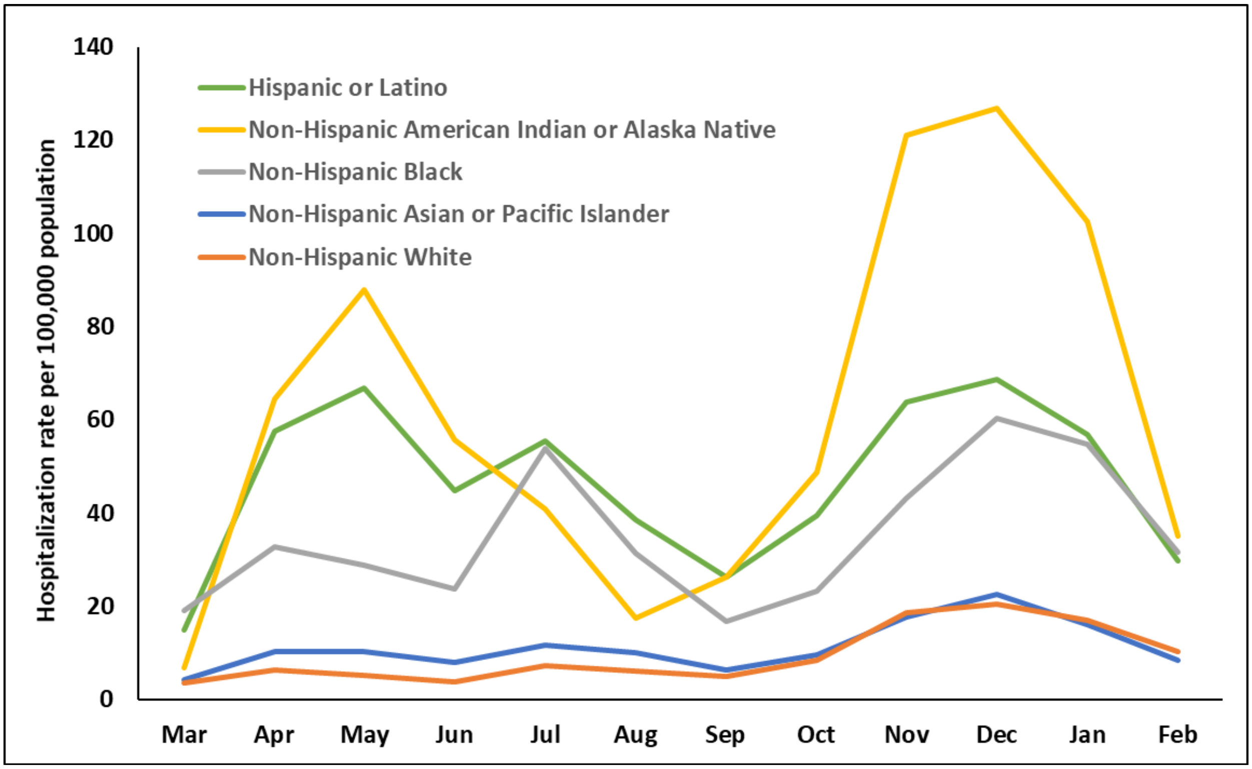

© 2021 Acosta AM et al. *JAMA Network Open.*

C, Adults aged 50-64 years

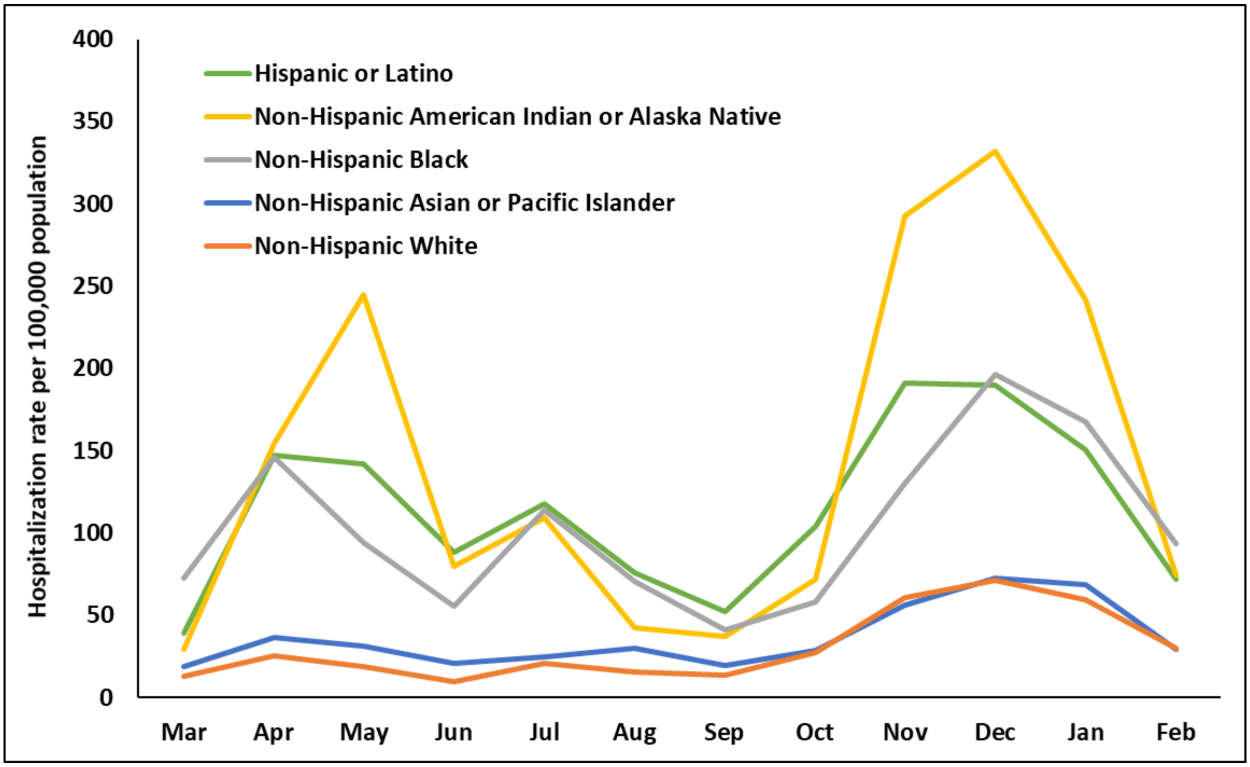

D, Adults aged ≥65 years

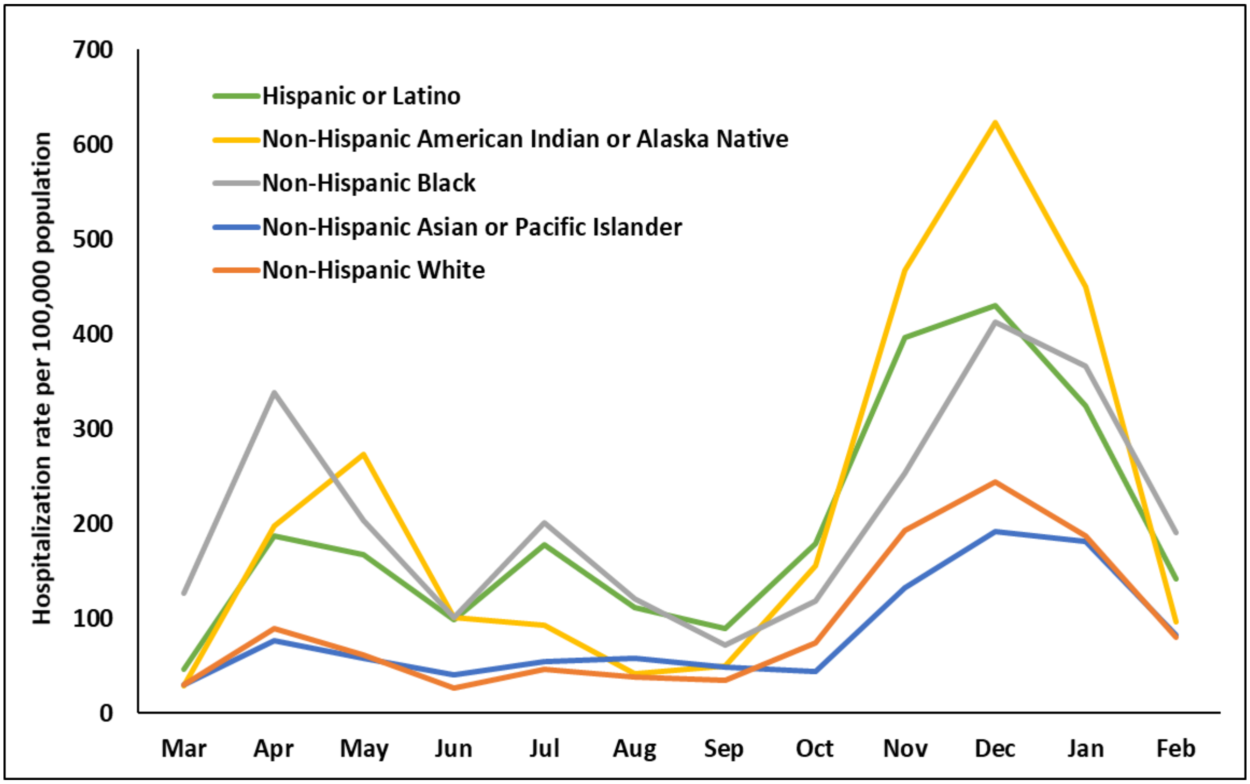

© 2021 Acosta AM et al. *JAMA Network Open.*

Supplemental Figure 2. Age-Adjusted Hospitalization, ICU Admission, and In-Hospital Mortality Rates by Race and Ethnicity and State, COVID-NET, March 1, 2020–February 28, 2021

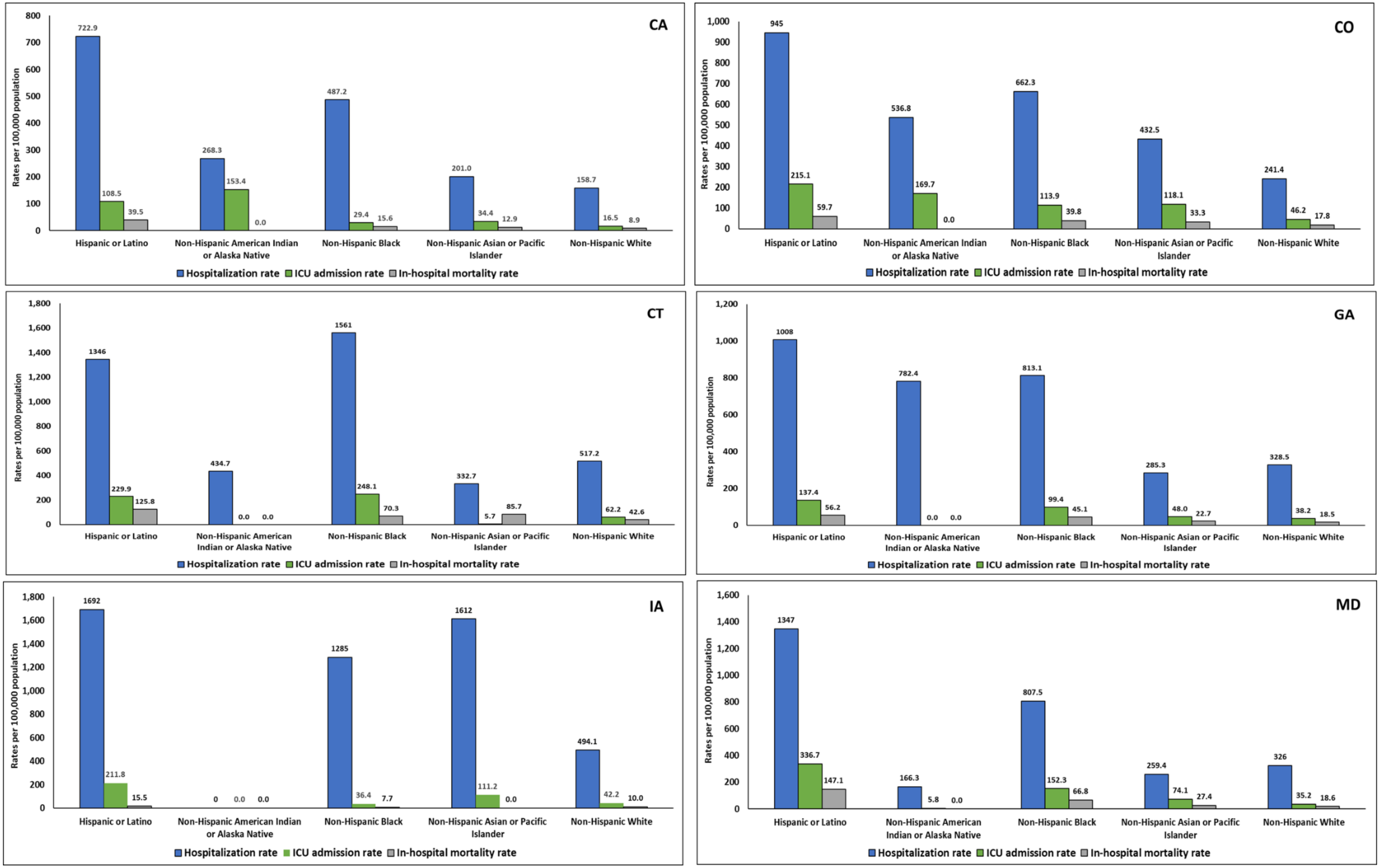

© 2021 Acosta AM et al. *JAMA Network Open.*

Supplemental Figure 2 continued…

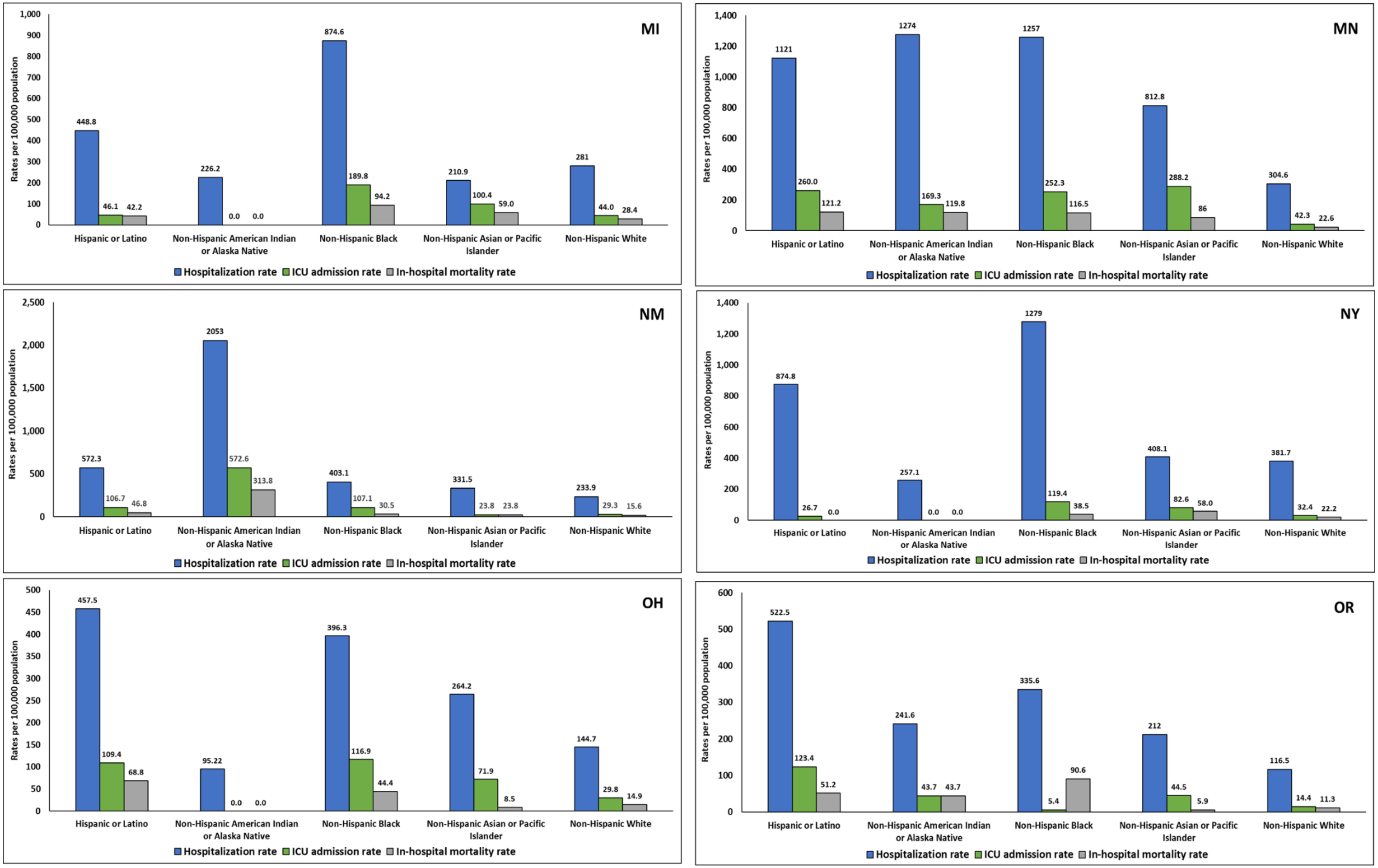

© 2021 Acosta AM et al. *JAMA Network Open.*

Supplemental Figure 2 continued…

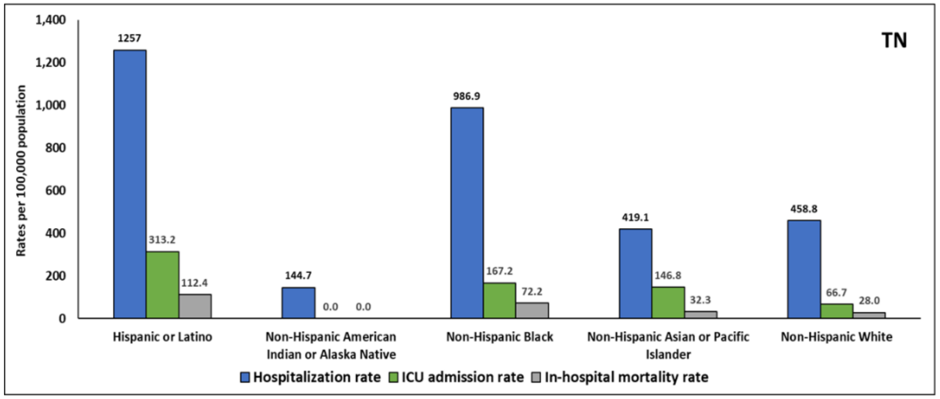

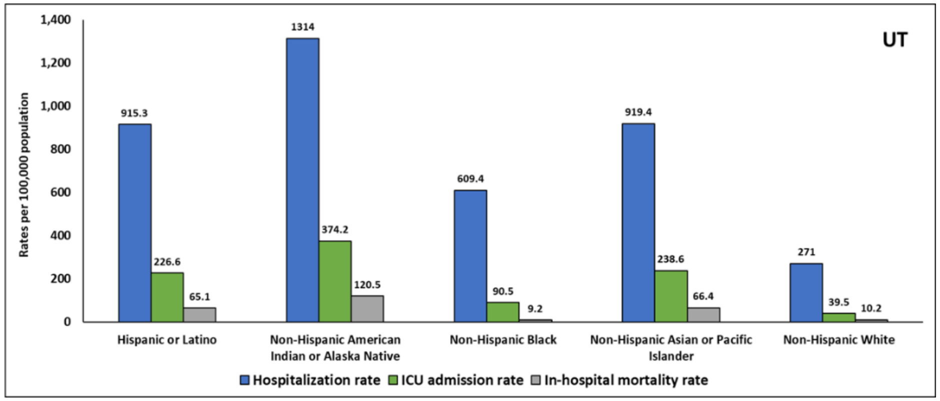

*Rates of 0.0 indicate 0 patients within that strata of race and ethnicity and state

© 2021 Acosta AM et al. *JAMA Network Open.*
